# Dynamics of the gut microbiota in rats after hypobaric hypoxia exposure

**DOI:** 10.7717/peerj.14090

**Published:** 2022-10-07

**Authors:** Yang Han, Jiayu Xu, Yan Yan, Xiaojing Zhao

**Affiliations:** 1 Translational Medical Research Center, Medical Innovation Research Division of Chinese PLA General Hospital, Beijing, China; 2 Key Laboratory of Biomedical Engineering and Translational Medicine, Ministry of Industry and Information Technology, Medical Innovation Research Division of Chinese PLA General Hospital, Beijing, China

**Keywords:** Gut microbiota, Dynamics, Hypobaric hypoxia, Normoxia, Anaerobes

## Abstract

**Background:**

Gut microbiota plays an important role in host health and is influenced by multiple factors. Hypobaric hypoxia usually existing at high altitude conditions can adversely affect normal physiological functions. However, the dynamic changes of gut microbiota influenced by hypobaric hypoxia have not been elucidated.

**Methods:**

In this study, we collected fecal samples from seven rats at 14 time points from entering the hypobaric chamber (eight time points) to leaving the chamber (six time points) and five rats served as normoxic controls. Metagenome sequencing was performed on all samples and the dynamics of taxa and functions were analyzed.

**Results:**

We found that the *α*-diversity was changed in the first 5 days after entering or leaving the hypobaric chamber. The *β*-diversity analysis revealed that gut microbiota structure was significantly separated among 14 time points. After entering the chamber, the relative abundance of *Bacteroides* decreased and the most abundant genus turned into *Prevotella*. The abundance of Firmicutes and Bacteroidetes showed an opposite trend and both have a significant change within 5 days after entering or leaving the hypobaric hypoxia chamber. Some obligate anaerobic bacteria belonging to *Desulfovibrio* and *Alistipes* were significantly enriched after entering the chamber for 5 weeks, whereas Probiotics like *Bifidobacterium* and *Lactococcus*, and short-chain fatty acids producers like *Butyrivibrio* and *Pseudobutyrivibrio* were significantly enriched after leaving the chamber for 3 weeks. Microbial functions like ‘Two-component regulatory system’, ‘beta-carotene biosynthesis’ and ‘Fatty acid biosynthesis’ were significantly enriched after entering the chamber for 5 weeks. Hypobaric hypoxia conditions could deeply affect the diversity and structure of gut microbiota. The alterations of abundance of dominant taxa (*Firmicutes* and *Bacteroidetes*), increased anaerobes and decreased probiotics induced by hypobaric hypoxia conditions might affect the host health.

## Background

Gut microbiota is closely related to host health. Currently, a large number of studies have revealed that gut microbiota is involved in various physiological activities such as immune defense (Strati et al., 2021), energy metabolism (Liu et al., 2020), and neural response (Morais, Schreiber & Mazmanian, 2020). In addition, gut microbiota are also disturbed by external factors like diseases (Fan & Pedersen, 2021), diets (Kolodziejczyk, Zheng & Elinav, 2019) and geography (Horwood et al., 2019). Hypoxia mainly occurs in a wide range of settings including high altitude and with some diseases like obstructive sleep apnea, chronic lung diseases and acute respiratory distress syndrome (Li et al., 2021). An ascent to high altitude regions usually results in hypoxia-related altitude illnesses such as acute mountain sickness, sleep disturbances and high-altitude headache (Li et al., 2021). However, most biological mechanisms are still not clear. Furthermore, more than 100 million people travel to high altitudes annually and over 140 million people have been living at high-altitude regions exceeding 2,500 m (Burtscher et al., 2001; Moore, 2001; West, 2015). Therefore, it is of great significance to explore the influences of hypoxia on gut microbiota.

At present, the effects of hypobaric hypoxia conditions on gut microbiota have been reported in a few studies. Hypobaric hypoxia increased the proportions of Enterobacteriales in gut microbiota of rats (Ramos-Romero et al., 2020). Intrauterine hypoxia changed the colonization of the gut microbiota in newborn rats (Sun et al., 2021). Short-term chronic intermittent hypobaric hypoxia alters gut microbiota composition in rats (Tian et al., 2018). Moreover, plateau hypoxia could cause alterations in the number and composition of intestinal microbes in rats (Zhang et al., 2018). In human, the hypoxia results in an altered diversity of gut microbial communities (Xing et al., 2018). These studies have indicated that hypobaric hypoxia conditions have a significant impact on the composition of gut microbiota in human and rats. However, these studies were based on cross-sectional research designs. Notably, various lines of evidence have indicated the gut microbiota is not static but, rather, a dynamic systems that constantly respond to external environmental and host-derived stimuli (Halfvarson et al., 2017; Smyth et al., 2020). Currently, there are not many studies longitudinally tracking changes in gut microbiota. Study on the dynamics of gut microbiota recovery after antibiotic exposure in young and old mice revealed the age was associated with differential recovery of the microbiota (Laubitz et al., 2021). Based on longitudinal investigation, the study revealed the dynamic image of the gut microbiota of mice after paramylon treatment (Taylor et al., 2020). The dynamics of changes in the gut microbiota of healthy mice fed with lactic acid bacteria and Bifidobacteria has also been explored (Gryaznova et al., 2022). However, dynamics of gut microbiota stimulated by hypobaric hypoxia and its recovery in rats have not been investigated.

In this study, we aimed to explore the dynamics of gut microbiota in rats at 14 time points during the process of entering the hypobaric chamber fed for 5 weeks and getting back to normoxic conditions fed for 3 weeks. Fecal samples were collected from 12 rats including seven rats for hypobaric hypoxia group (HH) from entering the hypobaric chamber (Inside, eight time points) to leaving the chamber (Outside, six time points) and five rats fed in normoxic conditions served as normal controls (NC) and metagenomic sequencing was performed. Study results showed the dynamics of the gut microbiota that was significantly disturbed by hypobaric hypoxia and anaerobes were significantly enriched in hypoxia conditions.

## Material and Methods

### Animal experiments

For the animal experiments, 10-week-old Sprague-Dawley (SD) male rats (*n* = 12) weighing 290 to 300 g were supplied by the Animal Center of the General Hospital of Chinese People’s Liberation Army (PLAGH). Animal experiments were performed strictly in accordance with the Guide for the Care and Use of Laboratory Animals published by the PLAGH. Rats were randomly assigned into the following two groups: the normal control group (NC, *n* = 5) and the hypobaric hypoxia group (HH, *n* = 7). Rats in the NC group were fed in normoxic conditions at an altitude of 43.5 m in Beijing and the samples from the NC group were collected only once. Rats in the HH group were fed in the hypobaric chamber (FLYDWC50-1C low pressure hypoxic experimental cabin produced by Guizhou Fenglei Air Ordance Co., Ltd. Guizhou, China) for 5 weeks (Inside group) and then returned to the normoxic conditions fed for 3 weeks (Outside group). The conditions of the hypobaric chamber were set to emulate an altitude of 5,500 m (50 KPa, 380 mmHg). In the Inside group, fecal samples at 1st day (In1d), 3rd day (In3d), 5th day (In5d), 1st week (In1w), 2nd week (In2w), 3rd week (In3w), 4th week (In4w) and 5th week (In5w) were collected sequentially. In the Outside group, fecal samples at 1st day (Out1d), 3rd day (Out3d), 5th day (Out5d), 1st week (Out1w), 2nd week (Out2w), 3rd week (Out3w) were collected sequentially. In the Inside group, fecal samples of three rats were removed due to contamination. In both groups, two rats were housed in a cage with a height of 30 cm and the floor of the cage covered with an adequate depth of dust free Aspen wood chip. To avoid coprophagy among rats, we isolated two rats housed in the same cage using wooden board. Playpens were provided in the cage for rats to exercise and socialize. The housed temperature and humidity were controlled at 25 ± 2 °C and 50 ± 10% with a 12 h light/12 h dark cycle. All rats have *ad libitum* access to standard pellet diet and water. Animals did need to be euthanized until the end of the experiment. If the rats are obviously unwell, the experiment should be terminated. We determined whether animals were unwell by observing behaviors such as silence, lethargy, inactivity, loss of appetite, and refusal to eat. After the experiment, all the animals were euthanized by intraperitoneal injection of barbiturates. All animal procedures and experimental protocols were reviewed and approved by the Animal Ethics Committee of PLAGH (2017-X13-05).

### Sample collection and DNA extraction

All fecal samples collected were frozen immediately at −80 °C within 20 min and transported to the laboratory with dry ice to extract bacteria DNA. DNA extraction was conducted at Novogene Bioinformatics Technology Co., Ltd. according to the standard protocols of Tiangen kits. The magnetic soil and stool DNA kit (TIANGEN®, Beijing, China) was used for DNA extraction. Special grinding beads were applied in this kit to effectively process the lysis of various complex components in fecal samples. The 0.25−0.5 g fecal samples were added to two mL centrifuge tube, and then 500 µL buffer SA, 100 µL buffer SC and 0.25 g grinding beads were added and mixed evenly with vortex. The mix was heated at 70 °C for 15 min to improve lysis efficiency, and then centrifuged at 12,000 rpm (∼13400 × g) for 1 min and the supernatant was transferred to a new two mL tube. 200 µL buffer SH was added and mixed with vortex for 5 s, and stood at 4 °C for 10 min. The mix was centrifuged at 12,000 rpm (∼13400 × g) for 3 min, the supernatant was transferred to a new two mL centrifuge tube, then 500 buffer GFA was added and mixed evenly. 10 µL of MagAttract Suspension G was added and mixed for 5 min. The centrifuge tube was placed on the magnetic stand for 30 s. After the beads were completely attached, the liquid in the tube was removed. Then, 700 µL of buffer RD was added and mixed for 5 min. The operation of placing the tube on the magnetic stand was repeated. Then, 700 µL of buffer PWD was added and mixed for 3 min and repeated once. After removing the liquid, the tube was placed on the magnetic stand and dried at the room temperature. Then, 80 µL of buffer TB was added and incubated at 56 °C for 5 min, and mixed three times, four times each time. After the beads were completely attached on the magnetic stand, the DNA solution was transferred and stored at −80 °C for subsequent sequencing analysis.

### Metagenomic sequencing and DNA library construction

All samples were paired-end sequenced on the Illumina platform at Novogene Bioinformatics Technology Co., Ltd. with insert size 350 bp and read length 150 bp. Preprocessing the raw data obtained from the Illumina HiSeq sequencing platform using Readfq (v8, https://github.com/cjfields/readfq) was conducted for quality control to acquire the clean data. Following quality control, removing reads containing low-quality bases (quality value ≤ 38) over 40 bp, ‘*N*’ bases over 10 bp and overlaps with the adaptor more than 15 bp. Also, the reads aligned to the human genome with Bowtie2 were removed (v2.2.4) (Langmead & Salzberg, 2012). The remaining clean reads were assembled by SOAP denovo (v2.04) (Luo et al., 2012) with several K-mer values (from 27 to 87), and the assembled results are interrupted from the ‘*N*’ junction to obtain Scaftigs without ‘*N*’ (Mende et al., 2012; Nielsen et al., 2014). All unmapped reads were combined for mixed assembly using the same methods and parameters as forward step. Scaftigs longer than 500 bp were retained for gene prediction using MetaGeneMark (v2.10). The unique initial gene catalogue was obtained by CD-HIT (v4.5.8) (Fu et al., 2012) with 95% identity and 90% minimum coverage. The abundance of each gene in each sample was calculated based on the number of aligned reads using Bowtie2.

### Taxonomy annotation and abundance profiling

In total, 3,096,427 de-redundant genes were used to assign taxonomic groups. Taxonomic assignments for genes were obtained by aligning the genes to integrated NR database (v 2018-01-02) of NCBI including Bacteria, Fungi, Archaea and Viruses using DIAMOND (v 0.9.9) (Buchfink, Xie & Huson, 2015) with the parameter setting are blastp, -e 1e−5. Taxonomic annotation of each gene was determined by the lowest common ancestor-based algorithm (LCA). The results of which the e value ≤ the smallest e value ×10 were chosen to take the LCA algorithm which was applied to system classification of MEGAN software to make sure the species annotation information of sequences (Oh et al., 2014). The abundance of a taxonomic level in each sample was calculated by summing the abundances of all genes annotated to that level and the gene number of a taxonomic level in each sample equals to the number of genes whose abundance was not zero.

### KEGG annotation and abundance profiling

The functional assignments of genes were acquired by aligning genes to Kyoto Encyclopedia of Genes and Genomes (KEGG) (Kanehisa et al., 2014) database using DIAMOND, and the highest-scoring annotated hits were retained for subsequent analysis. The abundance of each functional hierarchy was calculated by summing the abundance of genes annotated to that level.

### Microbial diversity and statistical analysis

The Shannon and Simpson diversity indexes (*α*-diversity) were calculated based on normalized species profiles by R package vegan (v2.5-6). *β*-diversity was analyzed by principal coordinate analysis (PCoA) by R package ape (v5.3) based on the Bray-Curtis distance. Dissimilarity analysis among groups was conducted by analysis of similarities (ANOSIM) by R package vegan (v2.5-6). Differential species or KEGG modules between Inside and Outside groups were identified using the MaAsLin2 by R package Maasline2 (v1.0.0). To remove the individual effect in longitudinal study, rat id was used as the random effect in differential analysis. Fold changes were the ratio of the average relative abundance of species or KEGG modules in Outside group and Inside group. The Benjamin-Hochberg false discovery rate (FDR) correction was performed for multiple hypothesis testing to compute FDR corrected *p*-values (q-values). Differential species or KEGG modules with *q*-value <0.05 were retained for subsequent analysis. Trend lines fitted using LOESS smoothing and the curve was plotted with a 95% pointwise confidence interval band in gray. A paired t test was performed, plotted and only one set of paired *t*-test was conducted at a time using GraphPad Prism version 8.3.1 (GraphPad Software, San Diego, California USA, www.graphpad.com).

### Correlation analysis

Correlation analysis was conducted for differential species and KEGG modules by R package psych (v2.0.7) based on Spearman’s rank correlation. Correlations with an absolute value of coefficient (r) >0.7 and *q*-value <0.05 were retained for further analysis and visualized by Cytoscape 3.7.2 (Shannon et al., 2003). Heat map was plotted by R package pheatmap (v1.0.12).

## Results

### Alterations of microbial community diversity and structure

**Figure 1:**
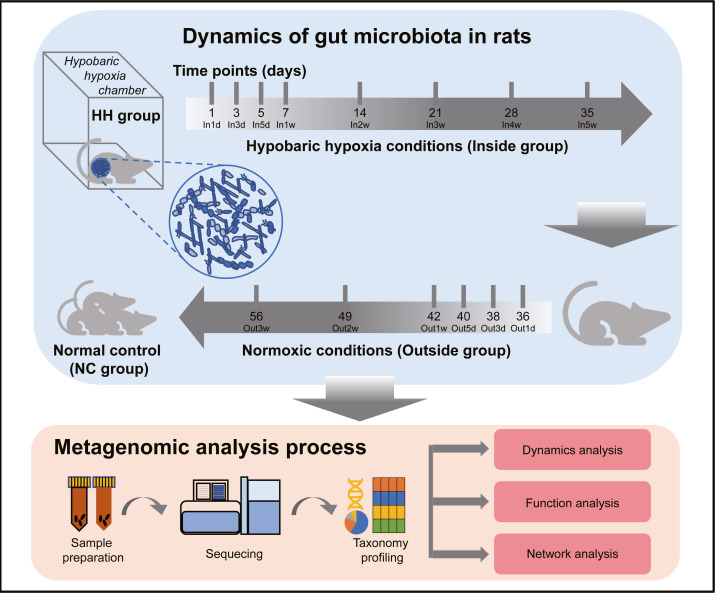
Experimental designs and metagenomic analysis process.

**Figure 2:**
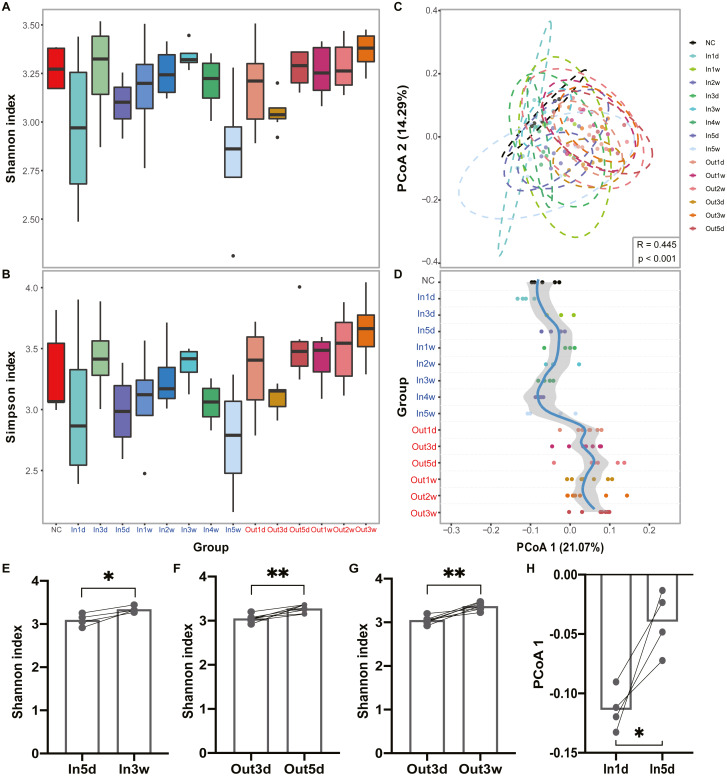
Diversity of gut microbiota was impacted by hypobaric hypoxia. (A) *α*-diversity of gut microbiota in each time point is measured with the Shannon index. (B) *α*-diversity of gut microbiota in each time point is measured with the Simpson index. (C) *β*-diversity of gut microbiota from samples collected in each time point is shown on a PCoA plot according to Bray-Curtis dissimilarity. (D). Samples across time points are plotted according to collection time on the *y*-axis, and the position on the *x*-axis is plotted according to first principal coordinate in PCoA. The color of the time points represents the group. Blue represents the Inside group and red represents the Outside group. Loess regression was applied to these points using the collection time points and the first principal coordinates, and the curve was plotted in blue with a 95% pointwise confidence interval band in gray. (E), (F) and (G). Paired test results of Shannon index between groups. (H). Paired t test results of PCoA 1 (21.07%) between In1d and In5d groups. ns *p* ≥ 0.05, * *p* < 0.05, ** *p* < 0.01, *** *p* < 0.001.

The experimental design and analysis processes of this study are presented in Fig. 1. To study the alterations of within-sample diversity (*α*-diversity) over time under hypobaric hypoxia conditions, Shannon and Simpson diversity index of all samples were calculated, respectively. Consistently, the Shannon and Simpson indexes both displayed similar trends over time (Figs. 2A and 2B). After entering the hypobaric chamber, we found that *α*-diversity had a significant fluctuation within the first 5 days after entering the chamber and then increased gradually and decreased after four weeks. To further statistically explore the fluctuation of diversity within the first 5 days and over time after entering or leaving the chamber, paired t test was performed between groups within the first 5 days and groups after that. Paired t test result showed that there is a significant difference in Shannon index between In5d and In3w (*p* = 0.02, Fig. 2E). After leaving the chamber, a significant fluctuation of *α*-diversity also occurred within the first 5 days and then increased gradually. Paired t test results also showed a significant difference in Shannon index between Out5d and Out3d (*p* = 0.002, Fig. 2F). Also, there was a significant difference between Out3w and Out3d (*p* = 0.001, Fig. 2G). The individual dynamics of *α*-diversity was presented in Figs. S1A and S1B. The *α*-diversity results indicated that the gut microbiota of rats responded strongly to hypobaric hypoxia or normoxia within 5 days. Furthermore, the overall community structure (*β*-diversity) across groups were examined by principal coordinates analysis (PCoA) based on Bray-Curtis distance (Fig. 2C). Significant differences were observed in the overall community structure (ANOSIM test, *R* = 0.445, *p* < 0.001), which indicated that there was a clear separation of microbial structure over time with the change of oxygen concentration and atmospheric pressure. The first principal coordinate in the Bray-Curtis PCoA showed the significant fluctuations of gut microbiota over time from entering the hyperbaric chamber to returning to the normoxic conditions (Fig. 2D). Paired t test showed a significant difference in the first principal coordinate between In1d and In5d (*p* = 0.02, Fig. 2H). The individual dynamics of the first coordinate in the Bray-Curtis PCoA was presented in Fig S1.

### Altered composition of gut microbiota over time

**Figure 3:**
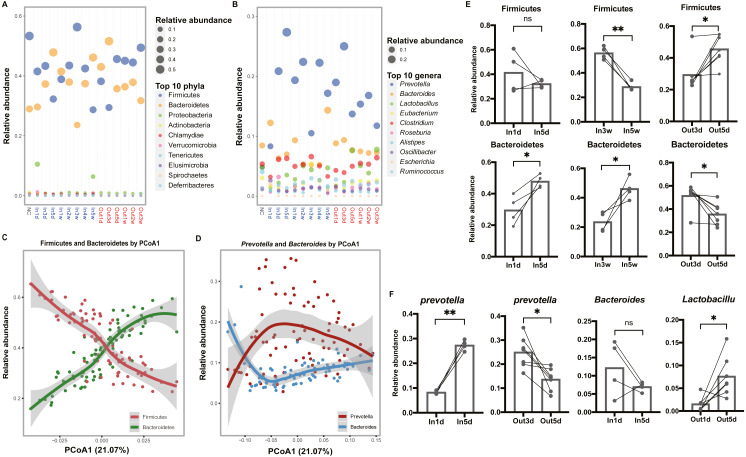
Composition of the top 10 phyla and genera in each group. (A) and (B) Relative abundance of the top 10 phyla and genera are plotted, respectively. The size of the point corresponds to the relative abundance value. The order of the phyla and genera in the legend from top to bottom represents the relative abundance from high to low. (C) and (D) Firmicutes and Bacteroidetes (*Prevotella* and *Bacteroides*) have an inverse relationship. Samples are plotted on the *y*-axis according to relative abundance of Firmicutes and Bacteroidetes (*Prevotella* and *Bacteroides*) versus the first principal coordinate of the original PCoA. (E) Paired t test results of the relative abundance of Firmicutes and Bacteroidetes between groups. (F) Paired t test results of the relative abundance of the *Prevotella*, *Bacteroides* and *Lactobacillus* between groups. ns *p* ≥ 0.05, *p < 0.05, **p < 0.01.

To explore the composition of gut microbiota over time, the top 10 abundant phyla and genera were screened out and visualized, respectively (Figs. 3A and 3B). In the phylum level, we observed that Firmicutes was more abundant than Bacteroidetes in the first three days after entering the hypobaric chamber, which was consistent with NC group. Paired t test result showed that the abundance of Bacteroidetes in In5d was significantly more abundant compared with In1d (*p* = 0.03, Fig. 3E). What’s more, from the second week to the fourth week, Firmicutes was more abundant than Bacteroidetes like initial state. Paired t test results showed that the abundance of Bacteroidetes in In5w was significantly more abundant compared with In3w (*p* = 0.04), while Firmicutes was reversed (*p* = 0.002, Fig. 3E). However, in the fifth week, the abundance of Bacteroidetes surpassed Firmicutes again. After leaving the chamber, the abundance of Bacteroidetes was the highest in the first three days. From the fifth day, the abundance of Firmicutes and Bacteroidetes reversed again, which was consistent with starting state and NC group. Paired t test result showed that the abundance of Bacteroidetes in Out3d was significantly more abundant compared with Out5d (*p* = 0.01), while Firmicutes was reversed (*p* = 0.01, Fig. 3E). The individual dynamics of relative abundance of Firmicutes and Bacteroidetes were displayed in Figs. S2A and S2B. These results indicated that normoxic conditions recovered the gut microbiota that destroyed by hypobaric hypoxia. Our results also revealed that Firmicutes was the most abundant phylum in normoxic conditions, and Bacteroidetes might was more adaptable to hypobaric hypoxia. Also, after entering or leaving the hypobaric chamber, the abundance of top 2 phyla both reversed on the fifth day, which implied that day 5 possibly was the time point when the composition of gut microbiota changed radically. In the genus level, we observed that the abundance of *Prevotella* was significantly increased after entering the hypobaric chamber and *Lactobacillus* was significantly enriched after leaving the chamber (Fig. 3B). The individual dynamics of relative abundance of top genera were displayed in Fig. S3. Paired t test result showed that the abundance of *Prevotella* was significantly increased In5d compared to In1d (*p* = 0.0005) and significantly decreased in Out5d compared to Out3d (*p* = 0.01, Fig. 3F). The abundance of *Lactobacillus* in Out5d was significantly increased compared with Out1d (*p* = 0.02, Fig. 3F). Previous study reported that Tibetan living at high altitude have a higher abundance of *Prevotella* whereas *Bacteroides* is more abundant in the Han living at the plain (Li et al., 2016). It implied that the *Prevotella* might be more adaptable to the high altitude with hypobaric hypoxia. Our results further prove the process of the abundance of *Prevotella* rising to the most abundant genus after exposure to the hypobaric hypoxia conditions. Although the abundance of *Prevotella* was decreased after leaving the hypobaric chamber five days later, it was still higher than *Bacteroides*. Previous study found that people living at high altitude maintained microbial signatures even after coming back to the plain three months later (Jia et al., 2020). Therefore, we speculated that it would take longer time to get back to the starting state.

To further investigate the relationships between dominant taxa, and their contributions to the separation of groups, loess regression was applied to points using the relative abundance and PCoA1 coordinates. We found that Firmicutes and Bacteroidetes showed an inverse trend (Fig. 3C). What’s more, the dominant genera, *Prevotella* and *Bacteroides*, had a similar relationship (Fig. 3D). These results further indicated that the competition between dominant taxa and the separation along the first principal coordinate was driven by a continuous variable that was a trade-off between *Prevotella* and *Bacteroides* (Gorvitovskaia, Holmes & Huse, 2016; Smits et al., 2017).

### Differential species between Inside and Outside groups

**Figure 4:**
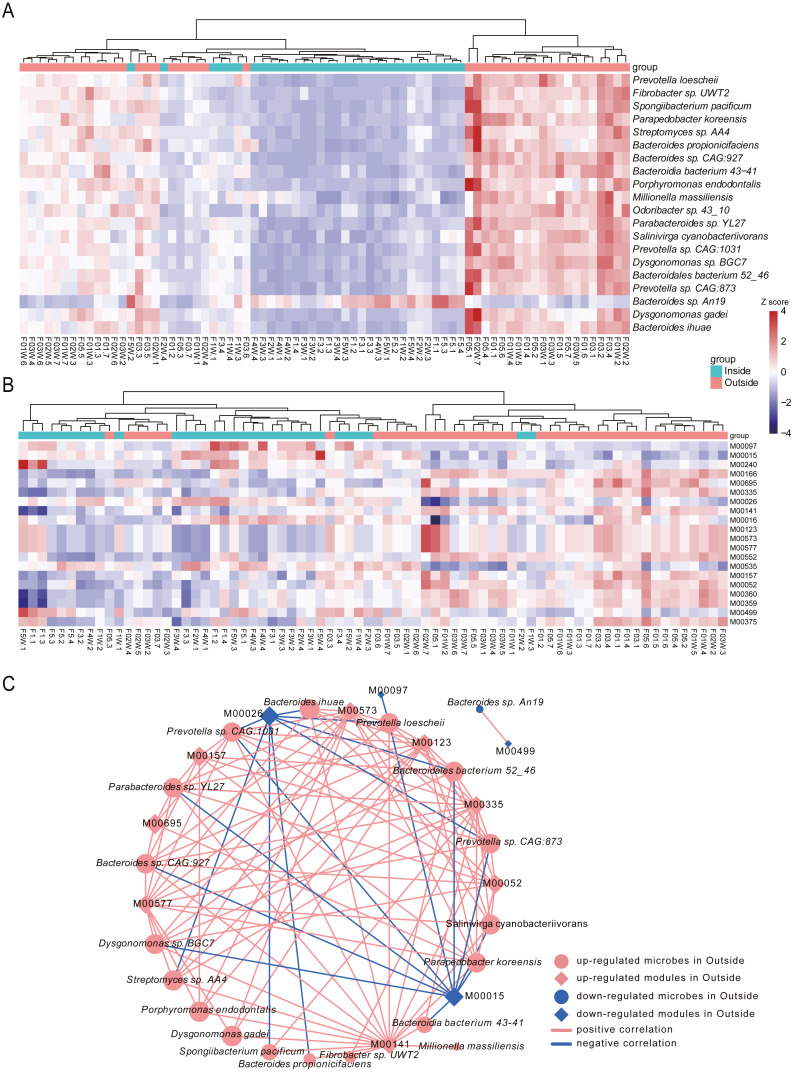
Differential species and KEGG modules between Inside and Outside groups. (A) Top 20 significantly differential species between Inside and Outside groups. (B) Top 20 significantly differential KEGG modules between Inside and Outside groups. In heat maps, the significance decreases from top to bottom. Heat maps were scaled by row and columns were clustered. (C) Correlations between top 20 differential species and top 20 KEGG modules with an absolute value of coefficient (*r*) > 0.7 and a statistical significance (*q*-value) < 0.05. Diamonds represent KEGG modules, circles represent bacteria. The size of the node is mapped to the degree of the node. Pink represents up-regulation in Outside group, and blue represents down-regulation in Outside group. Red lines represent the positive correlation, and blue lines represent the negative correlation.

To identify the species associated with hypobaric hypoxia, differential abundance analysis was performed between Inside and Outside groups using MaAsLin2. In total, 444 differential species were identified (*q*-value <0.05), in which 276 species were increased in Outside group and 168 species were increased in Inside group (Table S1). Differential species with top 20 significance were visualized with heat map (Fig. 4A).

In the top 20 significance species, 19 species were significantly enriched in Outside group. The abundance of *Prevotella loescheii*, which can ferment sugars to produce succinate and acetate, was the most significant species increased in Outside group. Also, *Butyrivibrio*, *Pseudobutyrivibrio*, *Porphyromonas endodontalis* and *Anaerophaga thermohalophila*, producing short-chain fatty acids (SCFAs) (Denger et al., 2002; Shah & Collins, 1988), were significantly enriched in the Outside group. Probiotics like *Bifidobacterium* and *Lactococcus* were significantly increased in Outside. Probiotics usually offer a large range of health benefits and have been indicated as a marker for health, thus their decreased abundance in Inside may be associated with some diseases like diarrhea induced by hypoxia (Gamah et al., 2021; Zheng et al., 2009). *Archangium gephyra* producing argyrin A, as a potent antitumoral drug (Nickeleit et al., 2008), was more abundant in Outside than that of Inside. *Veillonella atypica*, which improves run time *via* its metabolic conversion of exercise-induced lactate into propionate (Scheiman et al., 2019), was significantly increased in Outside group. In addition, some aerobes or facultative anaerobes like *Mycoplasma*, *Enterococcus* and *Paenibacillus* were significantly increased in Outside, while anaerobes like *Desulfovibrio* and *Alistipes* were decreased. As a result of their oxygen dependence, their increased or decreased abundance might be associated with the oxygen concentrations of Outside and Inside. The abundance of *Bacteroides vulgatus* and *Bacteroides dorei*, reducing gut microbial lipopolysaccharide production and inhibiting atherosclerosis (Yoshida et al., 2018), was significantly enriched in Inside group. *Oxalobacter formigenes*, regulating host oxalate homeostasis (Liu et al., 2017), was significantly increased in Inside group. *Bacteroides fragilis* and *Bacteroides caccae*, both are obligate anaerobes, were significantly enriched in Inside group. *Bacteroides massiliensis* enriched in obese children (Hollister et al., 2018), was also increased in Inside group.

### Microbial function analysis

To investigate the alterations of microbial function between Inside and Outside groups, differential analysis was performed for KEGG modules using MaAsLin2. In total, 96 KEGG modules with significant difference from 530 annotated modules were investigated (*q*-value <0.05, Table S2). Top 20 significantly differential modules were displayed with heat map (Fig. 4B). We found 32 modules were increased in Outside group, and 64 modules were decreased. In the top 20 differential modules, ‘beta-Carotene biosynthesis’ (M00097), and ‘Iron complex transport system’ (M00240) were significantly increased in Inside group. Pathways involved in biosynthesis of amino acids such as ‘Proline biosynthesis’ (M00015), ‘Histidine biosynthesis’ (M00026), ‘Lysine biosynthesis’ (M00016) and ‘Isoleucine biosynthesis’ (M00535) were significantly enriched in Inside group. ‘Two-component regulatory system’ (M00499, M00434, M00452, M00453, M00512), a basic stimulus–response coupling mechanism to sense and respond to changes of environmental conditions (Stock, Robinson & Goudreau, 2000), were significantly increased in Inside group. ‘Methanogenesis’ (M00567), an anaerobic respiration that generates methane as the final product of metabolism (Lyu et al., 2018), and ‘Fatty acid biosynthesis’ (M00083) were significantly increased in Inside group. In contrast, ‘cAMP signaling’ (M00695), ‘Reductive pentose phosphate cycle’ (M00166) and ‘Biotin biosynthesis’ (M00573) were significantly increased in Outside group.

To further investigate the correlation between top 20 significant species and microbial modules, Spearman rank correlation was conducted (Fig. 4C, Table S3). Correlation results showed that there are strongly significant correlations between 19 species and 12 modules (—r—>0.7, *q*-value <0.05). We found that many species enriched in Outside group were negatively correlated with ‘Proline biosynthesis’ (M00015) and ‘Histidine biosynthesis’ (M00026). However, ‘Biotin biosynthesis’ (M00573), ‘C1-unit interconversion’ (M00141) and ‘Pyrimidine ribonucleotide biosynthesis’ (M00052) were positively correlated with almost all species. Correlation results revealed that differential species influenced by hypobaric hypoxia conditions significantly facilitate the alterations of microbial functions.

## Discussion

Our findings highlight the gut microbiota in rats was significantly impacted by hypobaric hypoxia and dynamic changes over time from entering the hypobaric chamber to getting back to normoxic conditions. Study reported that the oxygen concentration in the gut of mice was decreased induced by intermittent hypoxia exposure (Moreno-Indias et al., 2015). What’s more, various evidences indicated both aerobic and facultative anaerobic bacteria colonized the intestine and consumed oxygen, allowing the increasing population of obligate anaerobes (Albenberg et al., 2014; Fanaro et al., 2003; Favier et al., 2002). Consistently, in Inside group, we found some anaerobic bacteria such as *Alistipes* and *Desulfovibrio*, were significantly enriched compared to Outside group. In contrast, some aerobes or facultative anaerobes like *Mycoplasma*, *Enterococcus* and *Paenibacillus* were significantly increased in Outside group. We speculated that the hypoxia or normoxia environment in which the hosts settle influenced the abundance of aerobes and anaerobes of gut. However, gut microbial sensing processes and pathways for hypoxia or normoxia need to be further explored. In addition, some enriched species in Inside group were related to diseases, such as *Bacteroides massiliensis* associated with promoting tumorigenesis (Garbas et al., 2021) and Bacteroides vulgatus markedly elevated in the gut microbiota of patients with polycystic ovary syndrome (Qi et al., 2019). Moreover, some probiotics and SCFA-producers like *Butyrivibrio*, *Bifidobacterium* and *Lactococcus* were significantly decreased in Inside group, thus leading to the symptoms of the digestive systems possibly (Gamah et al., 2021; Zheng et al., 2009). Our findings further provide the direct evidences that hypobaric hypoxia conditions have a profound impact on the gut microbiota. Previous studies have revealed the gut microbiota contributes to host physiology through the production of a myriad of metabolites (Krautkramer, Fan & Bäckhed, 2021). The composition and function of the gut microbiota in wild mammals were affected by high-altitude environments (Suzuki, Martins & Nachman, 2019). In addition, total aerobes at high altitudes were decreased significantly with increasing total facultative anaerobes by fecal microbiota analysis (Li & Zhao, 2015). Therefore, the alterations of gut microbiota induced by hypobaric hypoxia properly are related to host adaptation to the external environments.

Microbial functions involved in two-component regulatory system allowing organisms to sense and respond to changes in many different environmental conditions were significantly enriched in Inside group. Coherently, the increase of microbial functions about detecting physical and/or chemical changes in hypobaric hypoxia was beneficial for adaptation. Previous study found the activation of fatty acid biosynthesis maintains reduction potential and reduces lactoacidosis in neuronal cells under hypoxia thus facilitating adaptation to hypoxia (Brose, Golovko & Golovko, 2016). Our results also showed the significantly enriched ‘Fatty acid biosynthesis’ (M00083) in Inside group, which possibly was the same as that in mechanism. It was reported that beta-Carotene protected the body from oxidative stress damage (Sarada et al., 2002). The significant increase of ‘beta-Carotene biosynthesis’ (M00097) in Inside group might be a similar protective response to hypoxia-induced oxidative stress.

Dynamics of gut microbiota has been reported in many studies (Feng et al., 2020; Wang et al., 2020). To our knowledge, this is the first time to reveal the dynamic processes of gut microbiota in hypobaric hypoxia. Microbial diversity, structure and functions have continuous changes after exposure to hypobaric hypoxia. However, biological mechanism of dynamics and its recovery remain unclear. Keystone taxa associated with gut microbiome recovery after disruption are indispensable (Chng et al., 2020). In our study, the abundance of *Prevotella* and *Bacteroides*, both belonged to Bacteroidetes and were two abundant genera, were significantly impacted by concentration of oxygen. What’s more, the abundance of *Prevotella* and *Bacteroides* in samples showed opposite trends. We speculated that the two genera might are crucial to response to the change of oxygen concentration and their interplay promoted the dynamic changes of gut microbiota. Based on our results, more animal experiments are necessary to elucidate the biological mechanism.

## Conclusions

Our study provides a deep insight into the dynamics of gut microbiota in rats exposed to hypobaric hypoxia conditions. The significant fluctuations of *α*-diversity from hypobaric hypoxia to normoxic conditions and the PCoA results provided evidence for dynamics of gut microbiota over time. Firmicutes and Bacteroidetes (*Prevotella* and *Bacteroides*) showed significant changes from Inside to Outside groups, and their abundance presents a tendency to compete with each other. In hypobaric hypoxia, the enrichment of anaerobes and microbial pathways associated with environmental response and protecting against oxidative stress damage such as two-component regulatory system and beta-Carotene might be beneficial to adapting to hypobaric hypoxia.

##  Supplemental Information

10.7717/peerj.14090/supp-1 Figure S1 Individual diversity dynamics (A) and (B) Dynamics of *α*-diversity are displayed by individual. (C) Dynamics of PCoA 1 (21.07%) are presented by individual.

10.7717/peerj.14090/supp-2 Figure S2 Dynamics of the relative abundance of the top 2 phyla (A) Dynamics of the relative abundance of Firmicutes are displayed by individual. (B) Dynamics of the relative abundance of Bacteroidetes are displayed by individual.

10.7717/peerj.14090/supp-3 Figure S3 Dynamics of the relative abundance of the top 6 genera are displayed by individual

10.7717/peerj.14090/supp-4 Table S1 Differential species between Outside and Inside groups

10.7717/peerj.14090/supp-5 Table S2 Differential KEGG modules between Outside and Inside groups

10.7717/peerj.14090/supp-6 Table S3 Correlations between top 20 differential species and KEGG modules

10.7717/peerj.14090/supp-7 Supplemental Information 7 ARRIVE checklist

## Additional Information and Declarations

### DNA Deposition

The following information was supplied regarding the deposition of DNA sequences: All sequence data is available in the NCBI Sequence Read Archive: PRJNA734720.

### Data Availability

The following information was supplied regarding data availability: All sequence data is available in the NCBI Sequence Read Archive: PRJNA734720.

## References

[ref-1] Albenberg L, Esipova TV, Judge CP, Bittinger K, Chen J, Laughlin A, Grunberg S, Baldassano RN, Lewis JD, Li H, Thom SR, Bushman FD, Vinogradov SA, Wu GD (2014). Correlation between intraluminal oxygen gradient and radial partitioning of intestinal microbiota. Gastroenterology.

[ref-2] Brose SA, Golovko SA, Golovko MY (2016). Fatty acid biosynthesis inhibition increases reduction potential in neuronal cells under hypoxia. Frontiers in Neuroscience.

[ref-3] Buchfink B, Xie C, Huson DH (2015). Fast and sensitive protein alignment using DIAMOND. Nature Methods.

[ref-4] Burtscher M, Bachmann O, Hatzl T, Hotter B, Likar R, Philadelphy M, Nachbauer W (2001). Cardiopulmonary and metabolic responses in healthy elderly humans during a 1-week hiking programme at high altitude. European Journal of Applied Physiology.

[ref-5] Chng KR, Ghosh TS, Tan YH, Nandi T, Lee IR, Ng AHQ, Li C, Ravikrishnan A, Lim KM, Lye D, Barkham T, Raman K, Chen SL, Chai L, Young B, Gan YH, Nagarajan N (2020). Metagenome-wide association analysis identifies microbial determinants of post-antibiotic ecological recovery in the gut. Nature Ecology and Evolution.

[ref-6] Denger K, Warthmann R, Ludwig W, Schink B (2002). Anaerophaga thermohalophila gen. nov. sp. nov. a moderately thermohalophilic, strictly anaerobic fermentative bacterium. International Journal of Systematic and Evolutionary Microbiology.

[ref-7] Fan Y, Pedersen O (2021). Gut microbiota in human metabolic health and disease. Nature Reviews Microbiology.

[ref-8] Fanaro S, Chierici R, Guerrini P, Vigi V (2003). Intestinal microflora in early infancy: composition and development. Acta Paediatrica.

[ref-9] Favier CF, Vaughan EE, De Vos WM, Akkermans AD (2002). Molecular monitoring of succession of bacterial communities in human neonates. Applied and Environmental Microbiology.

[ref-10] Feng Q, Lan X, Ji X, Li M, Liu S, Xiong J, Yu Y, Liu Z, Xu Z, He L, Chen Y, Dong H, Chen P, Chen B, He K, Li Y (2020). Time series analysis of microbiome and metabolome at multiple body sites in steady long-term isolation confinement. Gut.

[ref-11] Fu L, Niu B, Zhu Z, Wu S, Li W (2012). CD-HIT: accelerated for clustering the next-generation sequencing data. Bioinformatics.

[ref-12] Gamah M, Alahdal M, Zhang Y, Zhou Y, Ji Q, Yuan Z, Han Y, Shen X, Ren Y, Zhang W (2021). High-altitude hypoxia exacerbates dextran sulfate sodium (DSS)-induced colitis by upregulating Th1 and Th17 lymphocytes. Bioengineered.

[ref-13] Garbas K, Zapała P, Zapała Ł, Radziszewski P (2021). The role of microbial factors in prostate cancer development-An Up-to-Date Review. Journal of Clinical Medicine.

[ref-14] Gorvitovskaia A, Holmes SP, Huse SM (2016). Interpreting prevotella and bacteroides as biomarkers of diet and lifestyle. Microbiome.

[ref-15] Gryaznova M, Dvoretskaya Y, Burakova I, Syromyatnikov M, Popov E, Kokina A, Mikhaylov E, Popov V (2022). Dynamics of changes in the gut microbiota of healthy mice fed with lactic acid bacteria and bifidobacteria. Microorganisms.

[ref-16] Halfvarson J, Brislawn CJ, Lamendella R, Vázquez-Baeza Y, Walters WA, Bramer LM, D’Amato M, Bonfiglio F, McDonald D, Gonzalez A, McClure EE, Dunklebarger MF, Knight R, Jansson JK (2017). Dynamics of the human gut microbiome in inflammatory bowel disease. Nature Microbiology.

[ref-17] Hollister EB, Foster BA, Dahdouli M, Ramirez J, Lai Z (2018). Characterization of the stool microbiome in hispanic preschool children by weight status and time. Childhood Obesity.

[ref-18] Horwood PF, Tarantola A, Goarant C, Matsui M, Klement E, Umezaki M, Navarro S, Greenhill AR (2019). Health challenges of the pacific region: insights from history, geography, social determinants, genetics, and the microbiome. Frontiers in Immunology.

[ref-19] Jia Z, Zhao X, Liu X, Zhao L, Jia Q, Shi J, Xu X, Hao L, Xu Z, Zhong Q, Yu K, Cui S, Chen H, Guo J, Li X, Han Y, Song X, Zhao C, Bo X, Tian Y, Wang W, Xie G, Feng Q, He K (2020). Impacts of the plateau environment on the gut microbiota and blood clinical indexes in han and tibetan individuals. mSystems.

[ref-20] Kanehisa M, Goto S, Sato Y, Kawashima M, Furumichi M, Tanabe M (2014). Data, information, knowledge and principle: back to metabolism in KEGG. Nucleic Acids Research.

[ref-21] Kolodziejczyk AA, Zheng D, Elinav E (2019). Diet-microbiota interactions and personalized nutrition. Nature Reviews Microbiology.

[ref-22] Krautkramer KA, Fan J, Bäckhed F (2021). Gut microbial metabolites as multi-kingdom intermediates. Nature Reviews Microbiology.

[ref-23] Langmead B, Salzberg SL (2012). Fast gapped-read alignment with Bowtie 2. Nature Methods.

[ref-24] Laubitz D, Typpo K, Midura-Kiela M, Brown C, Barberán A, Ghishan FK, Kiela PR (2021). Dynamics of gut microbiota recovery after antibiotic exposure in young and old mice (a pilot study). Microorganisms.

[ref-25] Li K, Dan Z, Gesang L, Wang H, Zhou Y, Du Y, Ren Y, Shi Y, Nie Y (2016). Comparative analysis of gut microbiota of native tibetan and han populations living at different altitudes. PLOS ONE.

[ref-26] Li L, Zhao X (2015). Comparative analyses of fecal microbiota in Tibetan and Chinese Han living at low or high altitude by barcoded 454 pyrosequencing. Scientific Reports.

[ref-27] Li X, Berg NK, Mills T, Zhang K, Eltzschig HK, Yuan X (2021). Adenosine at the interphase of hypoxia and inflammation in lung injury. Frontiers in Immunology.

[ref-28] Liu M, Koh H, Kurtz ZD, Battaglia T, PeBenito A, Li H, Nazzal L, Blaser MJ (2017). Oxalobacter formigenes-associated host features and microbial community structures examined using the American Gut Project. Microbiome.

[ref-29] Liu Z, Dai X, Zhang H, Shi R, Hui Y, Jin X, Zhang W, Wang L, Wang Q, Wang D, Wang J, Tan X, Ren B, Liu X, Zhao T, Wang J, Pan J, Yuan T, Chu C, Lan L, Yin F, Cadenas E, Shi L, Zhao S, Liu X (2020). Gut microbiota mediates intermittent-fasting alleviation of diabetes-induced cognitive impairment. Nature Communications.

[ref-30] Luo R, Liu B, Xie Y, Li Z, Huang W, Yuan J, He G, Chen Y, Pan Q, Liu Y, Tang J, Wu G, Zhang H, Shi Y, Liu Y, Yu C, Wang B, Lu Y, Han C, Cheung DW, Yiu SM, Peng S, Xiaoqian Z, Liu G, Liao X, Li Y, Yang H, Wang J, Lam TW, Wang J (2012). SOAPdenovo2: an empirically improved memory-efficient short-read de novo assembler. Gigascience.

[ref-31] Lyu Z, Shao N, Akinyemi T, Whitman WB (2018). Methanogenesis. Current Biology.

[ref-32] Mende DR, Waller AS, Sunagawa S, Järvelin AI, Chan MM, Arumugam M, Raes J, Bork P (2012). Assessment of metagenomic assembly using simulated next generation sequencing data. PLOS ONE.

[ref-33] Moore LG (2001). Human genetic adaptation to high altitude. High Altitude Medicine & Biology.

[ref-34] Morais LH, Schreiber HLT, Mazmanian SK (2020). The gut microbiota-brain axis in behaviour and brain disorders. Nature Reviews Microbiology.

[ref-35] Moreno-Indias I, Torres M, Montserrat JM, Sanchez-Alcoholado L, Cardona F, Tinahones FJ, Gozal D, Poroyko VA, Navajas D, Queipo-Ortuño MI, Farré R (2015). Intermittent hypoxia alters gut microbiota diversity in a mouse model of sleep apnoea. European Respiratory Journal.

[ref-36] Nickeleit I, Zender S, Sasse F, Geffers R, Brandes G, Sörensen I, Steinmetz H, Kubicka S, Carlomagno T, Menche D, Gütgemann I, Buer J, Gossler A, Manns MP, Kalesse M, Frank R, Malek NP (2008). Argyrin a reveals a critical role for the tumor suppressor protein p27(kip1) in mediating antitumor activities in response to proteasome inhibition. Cancer Cell.

[ref-37] Nielsen HB, Almeida M, Juncker AS, Rasmussen S, Li J, Sunagawa S, Plichta DR, Gautier L, Pedersen AG, Le Chatelier E, Pelletier E, Bonde I, Nielsen T, Manichanh C, Arumugam M, Batto JM, Quintanilha Dos Santos MB, Blom N, Borruel N, Burgdorf KS, Boumezbeur F, Casellas F, Doré J, Dworzynski P, Guarner F, Hansen T, Hildebrand F, Kaas RS, Kennedy S, Kristiansen K, Kultima JR, Léonard P, Levenez F, Lund O, Moumen B, Le Paslier D, Pons N, Pedersen O, Prifti E, Qin J, Raes J, Sørensen S, Tap J, Tims S, Ussery DW, Yamada T, Renault P, Sicheritz-Ponten T, Bork P, Wang J, Brunak S, Ehrlich SD (2014). Identification and assembly of genomes and genetic elements in complex metagenomic samples without using reference genomes. Nature Biotechnology.

[ref-38] Oh J, Byrd AL, Deming C, Conlan S, Kong HH, Segre JA (2014). Biogeography and individuality shape function in the human skin metagenome. Nature.

[ref-39] Qi X, Yun C, Sun L, Xia J, Wu Q, Wang Y, Wang L, Zhang Y, Liang X, Wang L, Gonzalez FJ, Patterson AD, Liu H, Mu L, Zhou Z, Zhao Y, Li R, Liu P, Zhong C, Pang Y, Jiang C, Qiao J (2019). Gut microbiota-bile acid-interleukin-22 axis orchestrates polycystic ovary syndrome. Nature Medicine.

[ref-40] Ramos-Romero S, Santocildes G, Piñol Piñol D, Rosés C, Pagés T, Hereu M, Amézqueta S, Torrella JR, Torres JL, Viscor G (2020). Implication of gut microbiota in the physiology of rats intermittently exposed to cold and hypobaric hypoxia. PLOS ONE.

[ref-41] Sarada SK, Sairam M, Dipti P, Anju B, Pauline T, Kain AK, Sharma SK, Bagawat S, Ilavazhagan G, Kumar D (2002). Role of selenium in reducing hypoxia-induced oxidative stress: an *in vivo* study. Biomedicine & Pharmacotherapy.

[ref-42] Scheiman J, Luber JM, Chavkin TA, MacDonald T, Tung A, Pham LD, Wibowo MC, Wurth RC, Punthambaker S, Tierney BT, Yang Z, Hattab MW, Avila-Pacheco J, Clish CB, Lessard S, Church GM, Kostic AD (2019). Meta-omics analysis of elite athletes identifies a performance-enhancing microbe that functions via lactate metabolism. Nature Medicine.

[ref-43] Shah H, Collins MD (1988). Proposal for reclassification of bacteroides asaccharolyticus, bacteroides gingivalis, and bacteroides endodontalis in a new genus, porphyromonas. International Journal of Systematic and Evolutionary Microbiology.

[ref-44] Shannon P, Markiel A, Ozier O, Baliga NS, Wang JT, Ramage D, Amin N, Schwikowski B, Ideker T (2003). Cytoscape: a software environment for integrated models of biomolecular interaction networks. Genome Research.

[ref-45] Smits SA, Leach J, Sonnenburg ED, Gonzalez CG, Lichtman JS, Reid G, Knight R, Manjurano A, Changalucha J, Elias JE, Dominguez-Bello MG, Sonnenburg JL (2017). Seasonal cycling in the gut microbiome of the Hadza hunter-gatherers of Tanzania. Science.

[ref-46] Smyth P, Zhang X, Ning Z, Mayne J, Moore JI, Walker K, Lavallée-Adam M, Figeys D (2020). Studying the temporal dynamics of the gut microbiota using metabolic stable isotope labeling and metaproteomics. Analytical Chemistry.

[ref-47] Stock AM, Robinson VL, Goudreau PN (2000). Two-component signal transduction. Annual Review of Biochemistry.

[ref-48] Strati F, Pujolassos M, Burrello C, Giuffrè MR, Lattanzi G, Caprioli F, Troisi J, Facciotti F (2021). Antibiotic-associated dysbiosis affects the ability of the gut microbiota to control intestinal inflammation upon fecal microbiota transplantation in experimental colitis models. Microbiome.

[ref-49] Sun Y, Li L, Song J, Mao W, Xiao K, Jiang C (2021). Intrauterine hypoxia changed the colonization of the gut microbiota in newborn rats. Frontiers in Pediatrics.

[ref-50] Suzuki TA, Martins FM, Nachman MW (2019). Altitudinal variation of the gut microbiota in wild house mice. Molecular Ecology.

[ref-51] Taylor HB, Gudi R, Brown R, Vasu C (2020). Dynamics of Structural and Functional Changes in Gut Microbiota during Treatment with a Microalgal *β*-Glucan, Paramylon and the Impact on Gut Inflammation. Nutrients.

[ref-52] Tian YM, Guan Y, Tian SY, Yuan F, Zhang L, Zhang Y (2018). Short-term chronic intermittent hypobaric hypoxia alters gut microbiota composition in rats. Biomedical and Environmental Sciences.

[ref-53] Wang J, Jia Z, Zhang B, Peng L, Zhao F (2020). Tracing the accumulation of *in vivo* human oral microbiota elucidates microbial community dynamics at the gateway to the GI tract. Gut.

[ref-54] West JB (2015). High-altitude medicine. The Lancet Respiratory Medicine.

[ref-55] Xing J, Ying Y, Mao C, Liu Y, Wang T, Zhao Q, Zhang X, Yan F, Zhang H (2018). Hypoxia induces senescence of bone marrow mesenchymal stem cells via altered gut microbiota. Nature Communications.

[ref-56] Yoshida N, Emoto T, Yamashita T, Watanabe H, Hayashi T, Tabata T, Hoshi N, Hatano N, Ozawa G, Sasaki N, Mizoguchi T, Amin HZ, Hirota Y, Ogawa W, Yamada T, Hirata KI (2018). Bacteroides vulgatus and bacteroides dorei reduce gut microbial lipopolysaccharide production and inhibit atherosclerosis. Circulation.

[ref-57] Zhang J, Chen Y, Sun Y, Wang R, Zhang J, Jia Z (2018). Plateau hypoxia attenuates the metabolic activity of intestinal flora to enhance the bioavailability of nifedipine. Drug Delivery.

[ref-58] Zheng W, Kuhlicke J, Jäckel K, Eltzschig HK, Singh A, Sjöblom M, Riederer B, Weinhold C, Seidler U, Colgan SP, Karhausen J (2009). Hypoxia inducible factor-1 (HIF-1)-mediated repression of cystic fibrosis transmembrane conductance regulator (CFTR) in the intestinal epithelium. The FASEB Journal.

