# Supplementary figures and images for: Dynamics of the gut microbiota in rats after hypobaric hypoxia exposure
Source: PeerJ. 2022 Oct 7;10:e14090. doi: 10.7717/peerj.14090 (PMC9549897; doi:10.7717/peerj.14090)

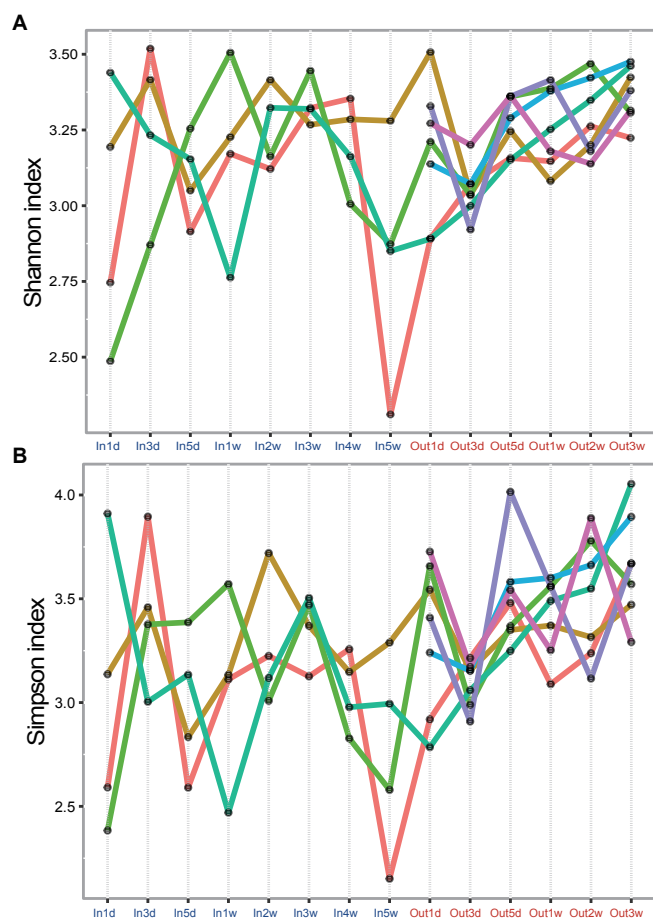

Rat id

- F.1
- F.2
- F.3
- F.4
- F.5
- F.6
- F.7

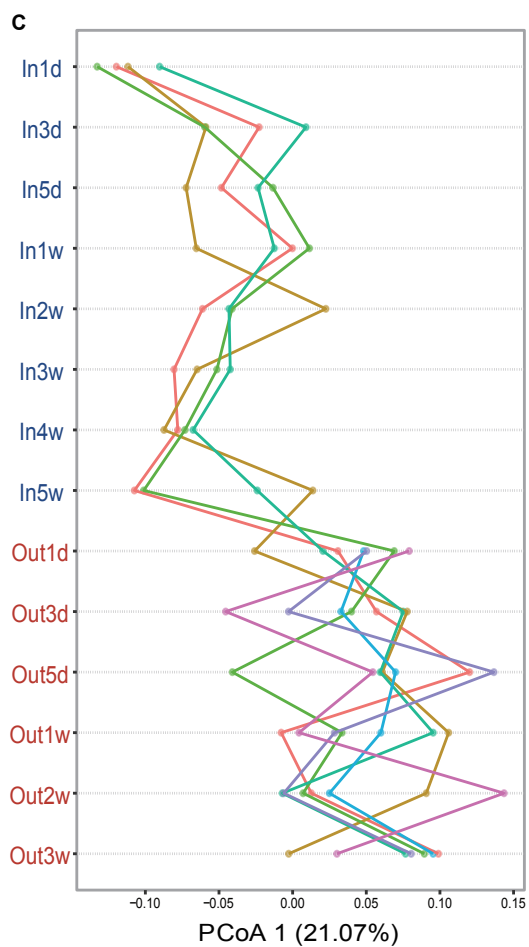

Supplement: Figure S1 — (A) and (B) Dynamics of α-diversity are displayed by individual. (C) Dynamics of PCoA 1 (21.07%) are presented by individual. [file peerj-10-14090-s001.pdf]

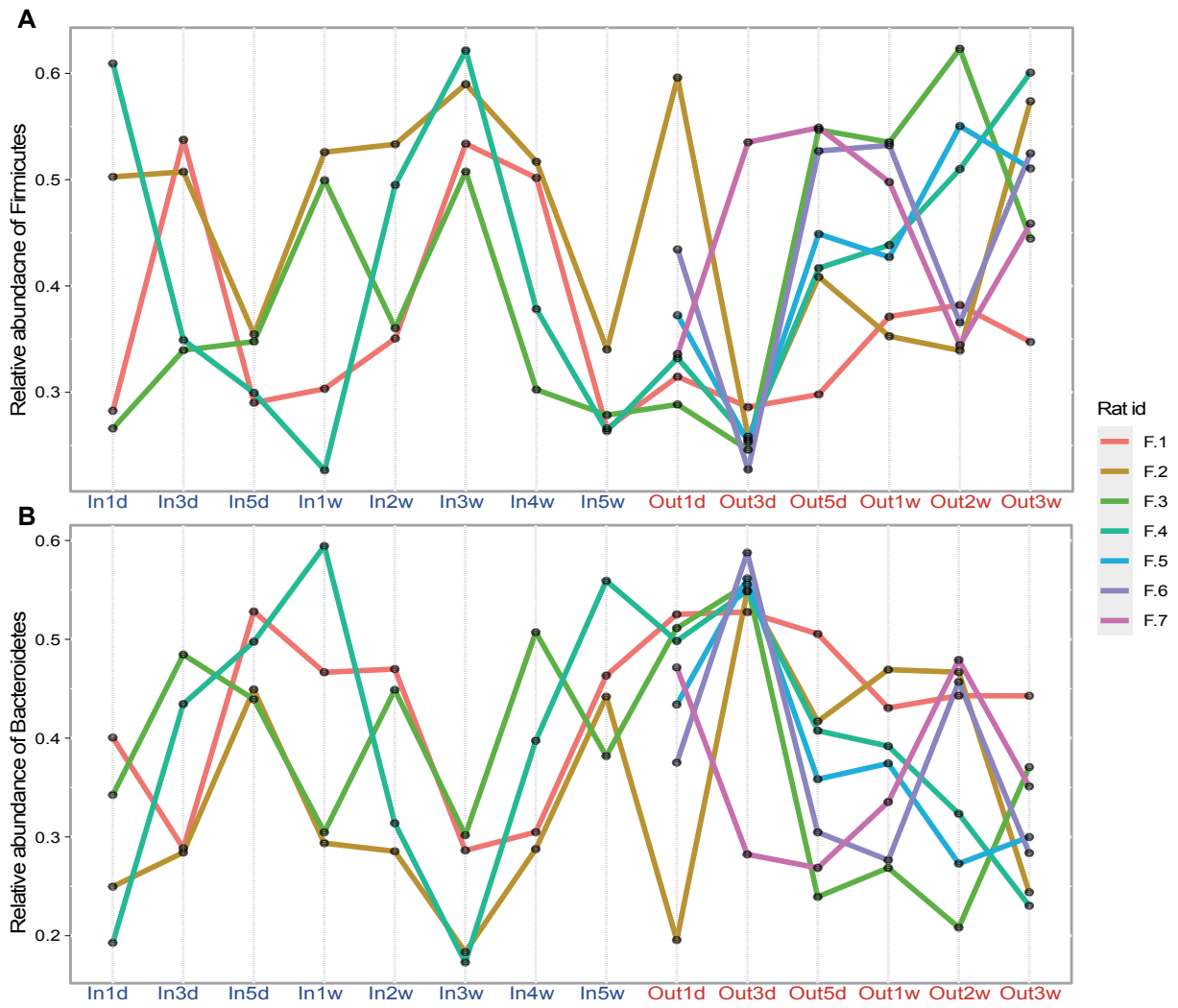

Supplement: Figure S2 — (A) Dynamics of the relative abundance of Firmicutes are displayed by individual. (B) Dynamics of the relative abundance of Bacteroidetes are displayed by individual. [file peerj-10-14090-s002.pdf]

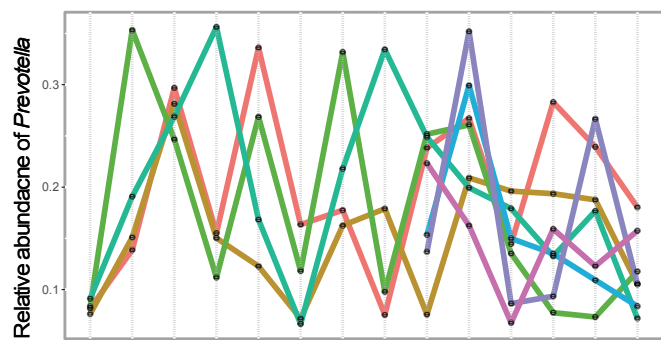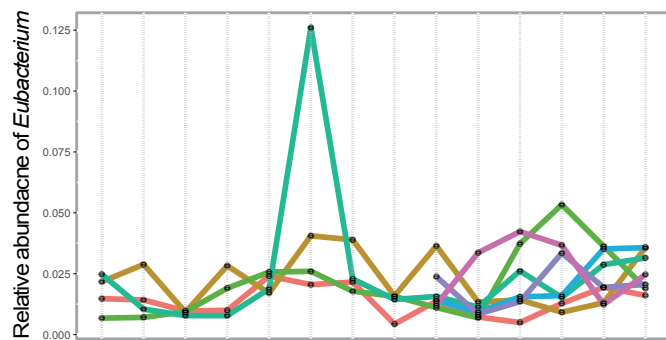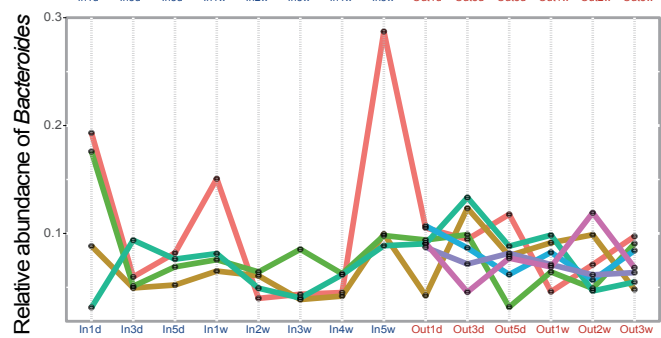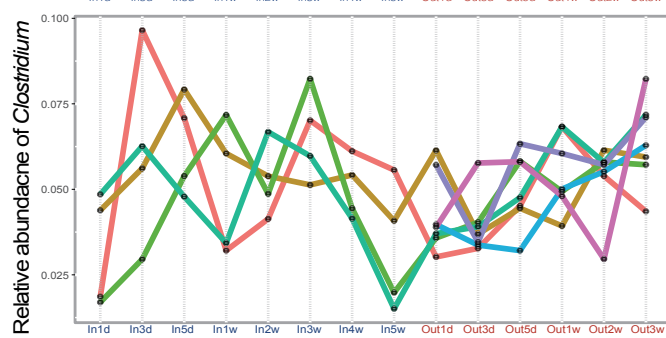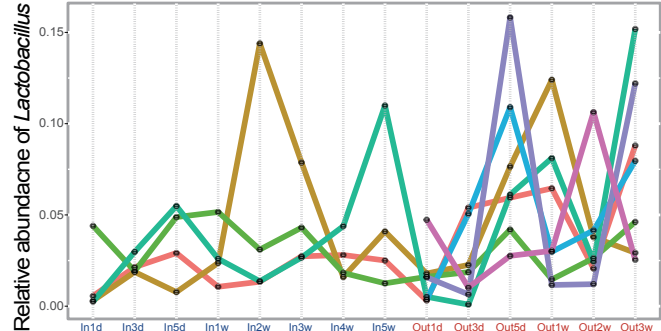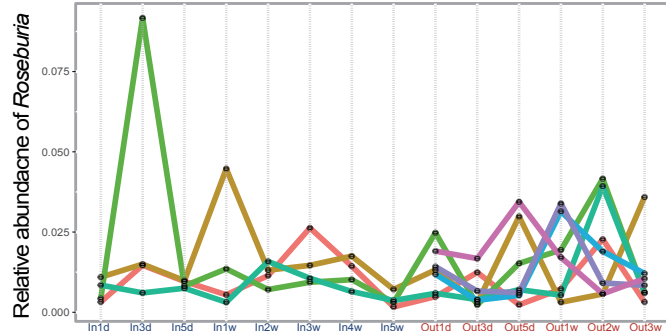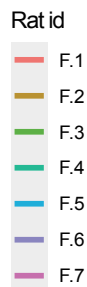

Supplement: Figure S3 [file peerj-10-14090-s003.pdf]
